# The effects of the Mulligan Sustained Natural Apophyseal Glide (SNAG) mobilisation in the lumbar flexion range of asymptomatic subjects as measured by the Zebris CMS20 3-D motion analysis system

**DOI:** 10.1186/1471-2474-9-131

**Published:** 2008-10-01

**Authors:** Maria Moutzouri, Evdokia Billis, Nikolaos Strimpakos, Polixeni Kottika, Jacqueline A Oldham

**Affiliations:** 1 Clinical Physiotherapist, Filoktitis Rehabilitation Center, Athens, Greece; 2 Centre for Rehabilitation Science, ARC Epidemiology Unit, School of Translational Medicine-Epidemiology Research Group, University of Manchester, Stopford Building, 2nd Floor, Oxford Road, Manchester, M13 9PT, UK; 3 Department of Physiotherapy, Technological Educational Institute (T.E.I.) of Patras, Branch Department of Aigion, Aigion 25100, Greece; 4 Department of Physiotherapy, Technological Educational Institute (T.E.I.) of Lamia, 35100, Greece; 5 Clinical Physiotherapist, Asimakopoulou 22, Patras, Greece

## Abstract

**Background:**

Mulligan's mobilisation techniques are thought to increase the range of movement (ROM) in patients with low back pain. The primary aim of this study was to investigate the application of the Mulligan's Sustained Natural Apophyseal Glide (SNAG) technique on lumbar flexion ROM. The secondary aim was to measure the intra- and inter-day reliability of lumbar ROM employing the same procedure.

**Methods:**

49 asymptomatic volunteers participated in this double-blinded study. Subjects were randomly assigned to receive either SNAG mobilisation (n = 25), or a sham mobilisation (n = 24). The SNAG technique was applied at the L_3_and L_4 _spinal levels with active flexion in sitting by an experienced manual therapist. Three sets of 10 repetitions at each of the two spinal levels were performed. The sham mobilisation was similar to the SNAG but did not apply the appropriate direction or force. Lumbar ROM was measured by a three dimensional electronic goniometer (Zebris CMS20), before and after each technique. For the reliability, five measurements in two different days (one week apart) were performed in 20 healthy subjects.

**Results:**

When both interventions were compared, independent *t *tests yielded no statistically significant results in ROM between groups (p = 0.673). Furthermore no significant within group differences were observed: SNAG (p = 0.842), sham (p = 0.169). Intra- and inter-day reliability of flexion measurements was high (ICC_1,1 _> 0.82, SEM < 4.0°, SDD<16.3%) indicating acceptable clinical applicability.

**Conclusion:**

While the Zebris proved to be a reliable device for measuring lumbar flexion ROM, SNAG mobilisation did not demonstrate significant differences in flexion ROM when compared to sham mobilisation.

**Trial registration:**

Current Controlled Trials NCT00678093.

## Background

It is common to find stiffness and reduced lumbar range of movement (ROM) in clinical presentations of mechanical low back pain (LBP) with a limited ability to perform flexion of the trunk [1-5]. Mulligan's mobilization-with-movement (MWM) treatment techniques are gaining increasing popularity for use in musculoskeletal conditions, such as low back pain (LBP) and other disorders [6-12]. One of the most important MWM technique is described as the SNAG, pioneered by Brian Mulligan [13]. SNAG is an acronym for "sustained natural apophyseal glide" with the technique described as involving the application of an accessory passive glide to the lumbar vertebrae while the patient simultaneously performs an active movement [13-15]. The direction of the glide is argued to be along the plane of the facet joints and the technique is performed in a weight-bearing position (i.e. sitting, standing).

Among the SNAG's basic principles of clinical management is an immediate reduction or cessation of pain and an increase in range of motion (ROM) [13-15]. The mechanism by which this MWM exerts its ameliorative effects in clinical practice remains somewhat of an enigma. According to Mulligan, the effect of MWMs is based on the premise that pain is associated with 'positional fault(s)' in joints with resultant subtle "biomechanical" changes [13,14] such as joint restriction and stiffness. Combining this joint glide with a physiological spinal movement (i.e. lumbar flexion), is argued to overcome the (biomechanical) joint problems that may be the cause of symptoms [14,15]. Despite this assertion, only one study, by Konstantinou et al. [16] has so far investigated the effects of MWM techniques on lumbar ROM amongst a LBP sample. The study reported statistically but not clinically significant results, as there is little information on the degree of change needed to constitute a real difference on lumbar mobility following the SNAG and therefore, the difference of interest was set at 7° (indicated as the average measurement error for the equipment) [16]. Thus, it is evident that further research is needed to confirm if ROM is influenced by applying the SNAG to the lumbar spine.

Measurement of ROM as an outcome measure for LBP patients is a common procedure both in clinical practice and in research providing clinicians with insight into the patient's overall physical profile [17-22]. ROM is either measured by routine clinical tests with limited validity and reliability [21-23], or by more accurate yet time-consuming laboratory methods. These include goniometers and inclinometers [24-27] with advantages and disadvantages in terms of their reliability and validity, as well as more sophisticated equipment such as the Lumbar Motion Monitor [19,28], the CA-6000 Spinal Motion Analyzer [29,30], the Fastrak Polhemus [31], and the Zebris [32], most of which have good reliability [33]. However, if normative values for spinal mobility are to be established, they must be considered device-specific [33]. Zebris was selected as an equipment measure of choice because of its ease of application as a portable apparatus with light miniature transmitters attached to the subject's body allowing unrestricted, comfortable motion [32,34]. However, although there are research studies demonstrating its validity and reliability in the cervical spine [34,35] and in scoliotic patients [31], there is no research to date measuring its reliability in the lumbar spine.

Given the above, the primary aim of this study was to explore whether an immediate increase in flexion ROM occurs following the application of a lumbar SNAG technique in an asymptomatic sample. A secondary aim was to investigate the reliability of the Zebris equipment utilized for measuring the lumbar ROM in the study.

## Methods

The intervention component of this study was a prospective randomized double-blinded controlled study investigating the effect of SNAG on lumbar flexion ROM of a healthy sample of volunteers. A reliability study of measuring lumbar flexion ROM with the three dimensional (3-D) electronic goniometer Zebris CMS20, preceded the main study. Ethical approval was obtained from the Ethical Committee of the Technological Educational Institute (T.E.I.) of Lamia, Greece.

Sample size was estimated using the methodology described by Howell [35], and Cohen [35] based on a mean difference in flexion ROM of 6.5 ± 12° derived from published lumbar ROM literature [16,36-40]. This is well above the upper limit of the 95% confidence interval of the standard error of measurement found in our reliability study, and gave us an estimated effect size of *d *= 3.8 (based on the formula indicated by Howell [34]). Thus, we aimed for a power estimate of 0.97 and to reach that level of power with our estimated effect size, we used 49 participants for the study.

### Sample

Forty-nine asymptomatic volunteers (20 males, 29 females) were recruited into the interventional component of the study. Their ages ranged between 18 and 32 years old (mean: 21 years). Exclusion criteria included parameters that could influence ROM i.e. no current or previous (within the last 2 years) LBP episodes, no spinal surgery or other spinal pathology (i.e. spondylolisthesis, scoliosis) [41-45]. Moreover, subjects with serious vascular or heart problems, or subjects taking anticoagulants were excluded since such conditions can be implicated as contraindications for manual therapy [46,47]. For the reliability component, 20 volunteers (11 males, 9 females) participated in the study, with ages ranging between 19 and 23 years old (mean: 20,7 years). All subjects signed an informed consent form before participation.

### Equipment

**Figure 1:**
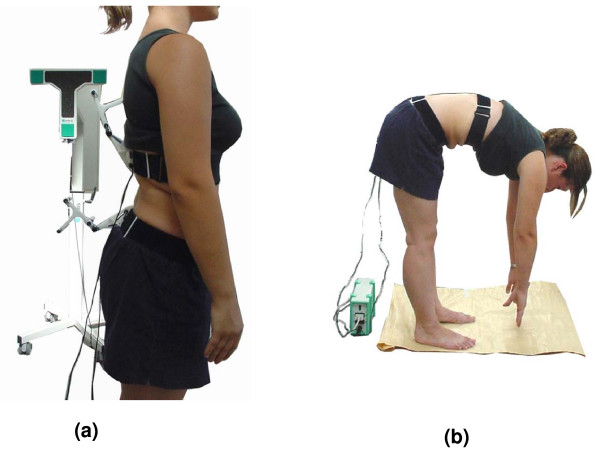
**Measurement with the Zebris**. Starting position; the miniature transmitters (on the subject's velcro traps) and the transmitting stand are seen (a). Measurement of flexion ROM with participant standing on marked tracks (b).

Lumbar ROM was measured with the Zebris CMS20 ultrasound-based motion analysis system (Winspine, triple lumbar, Zebris Medizintecnik GmbH, Isny Germany). This 3-D lumbar motion equipment consists of two specially designed belts (velcro bands adjustable to individual lumbar sizes) each attached to a series of three miniature ultrasound transmitters that are fixed to the subject's torso (Figure 1a). For isolating lumbar movement, one belt was placed firmly around the T12 lumbar level and the other around the level of the posterior superior iliac spines (PSISs) and the anterior superior iliac spines (ASISs). Measurement was based on determination of the spatial coordinates of the ultrasound transmitters by a fixed system of three microphones which was positioned at a special stand close by. Using triangulation, the measurement was derived from the time delay between the ultrasound pulses measured at a sampling rate of 20 Hz. The spatial position of the lumbar spine was calculated by the system's software [48-51].

### Measurements of lumbar flexion

ROM measurements were performed by a physiotherapist experienced in the use of Zebris equipment. The therapist was blinded to the type of intervention. All measurements took place at least two hours after waking up in the morning in order to overcome the initial diurnal stiffness of the spine and circadian variation [31,52,53]. Foot position was standardised by drawing footprints on a piece of flipchart paper. Prior to the measurements, each participant performed 4–5 repetitions of lumbar flexion to warm-up in order to achieve consistency of flexion ROM [52,54,55]. Standardization of strap position was achieved by marking their borders on each subject's body while the subject assumed a flexed position. These were then fixed in place using adhesive tape. This flexed position of strap application was found to be the most 'stable' for preventing strap movement during measurement. Calibration took place with the subject assuming an upright position. Each participant was then asked to perform three repetitions of forward bending of their trunk as far as possible in their own preferred speed with their knees kept straight (Figure 1b). On completion, the physiotherapist recorded the lumbar ROM values. Following the manual intervention, another set of three lumbar flexion measurements was undertaken under the same standardized conditions.

### Application of the manual interventions

**Figure 2:**
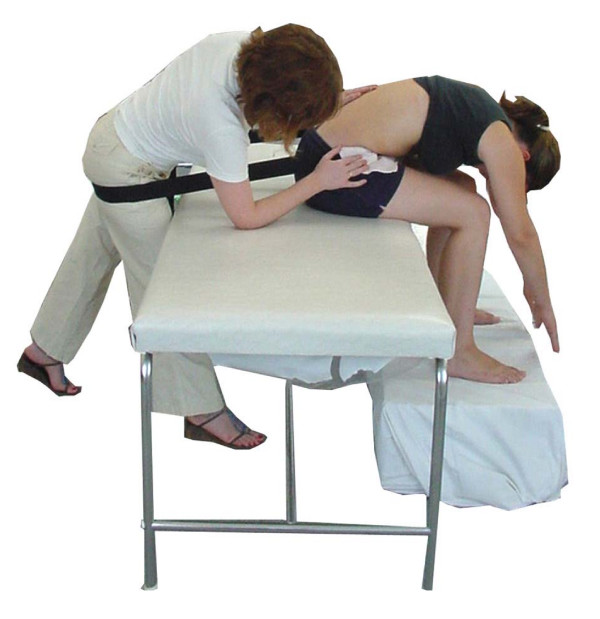
Application of the SNAG technique.

An accredited manual therapist with 7 years of clinical experience in treating LBP patients and substantial training in the Mulligan concept performed the manual techniques. Following the first set of ROM measurements, each participant was randomly assigned into one of the two intervention groups (SNAG or sham) by selecting one of two identical cards representing each technique. Neither the participant, nor the physiotherapist performing the measurements was aware of the selected technique. The techniques were applied by the manual therapist on L3 and L4 lumbar levels. These particular levels were chosen because it has been found in cadaver segmental motion studies that L4/5 has stiffer elements compared to other motion segments [56-59] and may account for limitations in ROM more often than other lumbar levels. Both techniques were performed from a comfortable sitting position over a plinth, with the participant's legs resting on an adjustable height stool (Fig. 2). SNAGs were then performed at these spinal levels with active lumbar flexion. Three sets of ten repetitions were performed following a test trial for familiarizing the subject with the technique and ensuring optimal comfort. A belt was used, as indicated by Mulligan [13], in order to stabilize the pelvic girdle and allow each subject to obtain full active flexion with ease. A manual force (according to the therapist's clinical judgment) was applied and sustained (during active flexion) in a direction argued to be parallel to the lumbar facet joints. This was applied by direct palpation via the ulnar border of the therapist's hand on the relevant spinous process in the manner described by Mulligan [13]. Each SNAG was sustained for a few seconds at the end of range of flexion.

The sham technique appeared very similar to the SNAG but the application of the optimum direction and the appropriate amount of force were absent. In particular, the therapist's hand was just placed (without exerting any pressure) over the relevant spinous process and the direction of this very 'gentle' pressure of the hand was downward (rather than parallel to the facet joint plane direction). These two factors (direction and force) are considered to play a vital role in the success of the technique [11,13]. None of the participants in either group expressed any discomfort at any point throughout the procedure. A two-minutes' break followed the intervention before reassessing ROM.

### Reliability procedure

For the reliability measurements, all procedures undertaken (standardised position, warm-up, instrument's calibration, application of the velcro straps etc.) were identical with the interventional component of the study. Each subject was examined on five occasions; with three tests were performed the same day (intra-day reliability), and the other two tests performed one-week later (inter-day reliability). On each occasion, following a practice trial, three active lumbar flexion movements were performed by each subject at their normal, preferred speed [50,54,60-62] and the mean of the three repetitions was recorded [48,54,60,61].

### Data analysis

As all data were normally distributed an independent sample test was utilized for detecting statistical differences in ROM between the groups (SNAG versus sham). The paired tests were utilized to explore statistical differences in lumbar ROM within each group. Levels of significance were set at 5%. For the reliability study, the intraclass correlation coefficient (ICC_1,1_), standard error of measurement (SEM) and the smallest detectable difference (SDD) reliability indices were used for all parameters being tested. The SDD represents a clinically applicable percentage figure for the amount of change needed to detect a true change in a subject's performance [63-65]. All data were analyzed using the Statistical Package for the Social Sciences (SPSS, Version 11.5).

## Results

Following the randomization procedure in order to allocate participants to the intervention groups, 25 participants were allocated to the SNAG group (12 men, 13 women) and 24 to the sham group (8 men, 16 women). No significant differences were noted between the groups' demographic and baseline ROM data (Table 1).

**Table 1:** Profile of the SNAG and sham groups.

	SNAG (n = 25)	Sham (n = 24)	p value	95% CI (Lower-Upper)
Male	12	8	0.306	-0.432 – 0.139
	mean (SD)	mean (SD)		
Age (years)	21.5 (2.5)	21.6 (2.5)	0.684	-1.18079 – 1.78413
Height (m)	1.70 (9.3)	1.69 (9.11)	0.624	-3.98971 – 6.57971
Weight (kg)	66.2 (12.4)	66.3 (12.6)	0.610	-5.43709 – 9.16375
BMI (kg/m^2^)	22.5 (2.6)	22.7 (2.7)	0.706	-1.27923 – 1.87323
ROM at baseline	63 (8.33)	60.69 (10.92)	0.382	-3.17391 – 8.12887

CI – Confidence Intervals, SD-standard deviation

For the ROM measurements, only the second and third recordings from each repetition were used in order to avoid any learning effect often observed between the first and second repetition [66]. A mean difference of 1.2° flexion was detected in favor of the SNAG intervention when compared to the sham group (Table 2) though this difference did not reach statistical significance (p = 0.673). Paired *t*-tests for lumbar ROM before and after the application of each technique, yielded no statistically significant differences within the SNAG (p = 0.842), nor the sham group (p = 0.169). Statistical results and confidence intervals (CI) following the interventions are illustrated in Table 3.

**Table 2:** Range of movement values at baseline and following each intervention.

	Flexion ROM (°)			
	SNAG (n = 25)		Sham (n = 24)	
	Before	After	Before	After
Mean (SD)	63 (8.3)	63.1 (7.9)	60.7 (10.9)	61.9 (11.1)
Minimum	49.6	50.0	34.9	40.2
Maximum	83.2	80.7	77.6	79.3

ROM – Range of movement

**Table 3:** Statistical differences between the intervention groups.

	P value (2-tailed)	95% CI (Lower-Upper)
Differences between groups after the interventions	0.673	-4.37868 – 6.72090
Differences within the SNAG group	0.842	-1.4227 – 1.1690
Differences within the sham group	0.169	-3.0872 – 0.5735
Gender differences	0.736	-0.7528 – 10.2122

CI – confidence intervals

For the reliability study, the anthropometric characteristics of the subjects are presented in Table 4. The ICCs ranged between 0.89 and 0.90 for the intra-day measurements, and was 0.82 for inter-day measurements, indicating excellent reliability. All reliability indices for intra-day and inter-day flexion measurements are summarized in Table 5.

**Table 4:** Anthropometric characteristics of subjects involved in the reliability study.

	Sample (n = 20)		Males (n = 11)		Females (n = 9)	
	Mean (SD)	Range	Mean (SD)	Range	Mean (SD)	Range
Age (years)	20.7 (1.0)	19–23	20.6 (1.0)	20–23	20.8 (1.1)	19–23
Height (cm)	172.9 (9.6)	159–191	178.5 (8.5)	165–191	166.1 (5.6)	159–175
Weight (kg)	66.6 (11.6)	47–87	74.5 (7.9)	60–87	57.0 (7.4)	47–70

SD: standard deviation

**Table 5:** Reliability values and paired sample *t *tests for intra-day and inter-day measurements.

Measurement	ICC_(1,1)_	SEM	SDD	95% CI (Lower-Upper)	p value (t test)	Mean
Intra-day (measured on Day 1)	0.89	3.3°	13.8%	0.79 – 0.95	0.60	67.4°
Intra-day (measured on Day 8)	0.90	3.0°	12.1%	0.77 – 0.96	0.35	68.5°
Inter-day (between the different days' measurements)	0.82	4.0°	16.3%	0.61 – 0.92	0.28	68.3°

ICC: intraclass correlation coefficient; SEM: standard error of measurement; SDD: smallest detectable difference (expressed as percentage of grand mean).

## Discussion

The study was designed to investigate the immediate mechanical effect of a Mulligan's SNAG technique on lumbar flexion ROM since it is suggested that flexion is the movement most often affected in LBP [1,2,24]. The findings did not yield statistically significant differences between SNAG and sham intervention. The slight increase that was noted post application of the SNAG technique was not considered to be clinically important since it lies within the limits of the measurement error of the lumbar ROM measurements (as indicated from the reliability study).

These results are in accordance with previous literature demonstrating non-statistically significant results in ROM when applying manipulative therapy techniques in asymptomatic subjects [67,68]. Petty et al [67] found no change in flexion ROM (recorded with the CA-6000 Spinal Motion Analyzer) after a 2-minute grade IV postero-anterior (PA) mobilization technique to L3, applied in a healthy sample for over 3 days. It could be argued that the amount of mobilization was not adequate to produce any change in healthy subjects. One randomized controlled trial however, found that a two-minute grade III passive physiological mobilization applied in flexion, in a healthy sample with less than average ROM, did produce an increase in flexion, as measured with finger-tip-to floor test [69]. The main difference between this latter and the current study was the use of a sample with reduced flexion ROM. However, it could also be argued that results could be influenced by the fact that participants had prior knowledge of being in receipt of the active intervention. Furthermore, the finger-tip-to-floor test has shown controversial results in terms of reliability [20,22]. The first study investigating the effect of lumbar spine PA mobilizations on ROM, stiffness and pain on a symptomatic population is a cross-over design by Goodsell [70]. LBP patients exhibiting pain on active flexion or extension were treated with grade III or IV PA glides on the most symptomatic lumbar level for three one-minute repetitions of mobilizations. Results indicated reduction in pain scores during active movement, without however, any differences in stiffness or ROM. Authors concluded that absence of change in the mechanical parameters (ROM and stiffness) was due to small changes in pain not sufficient to produce detectable differences. In addition, an order effect in this cross-over design cannot be precluded, since the control group received the intervention first; thus, indicating that by applying the placebo treatment to all patients *first *(rather than randomly applying placebo and intervention treatments), the study's outcome could have been affected (by this 'order').

So far, research regarding MWMs' effectiveness in increasing ROM is limited to case studies where only peripheral joints are evaluated [12,71]. Only one recent study by Kostantinou et al [16] investigated the immediate effects of MWM's in ROM and pain levels in 26 LBP patients with pain and flexion ROM limitations. The treatment consisted of SNAG mobilizations of 1 to 3 levels, using 2–3 sets of 4–6 repetitions (at each level), whereas, the placebo consisted of adoption of a comfortable position for around three minutes' time. Results indicated that 73% of the intervention condition and 35% of the placebo condition had improvements in flexion-extension ROM (as measured with an inclinometer) and/or pain scores. However, placebo administration, and a crossover design carries the risk of a residual effect from the intervention and could be a limiting factor. Nevertheless, the aforementioned study was the first investigating Mulligan MWM's effect in a symptomatic LBP population [16]. Given the above, it is questionable whether MWMs utilise purely biomechanical, or more complex underlying mechanisms and interactions in order to produce the claimed rapid restoration of pain-free movement. More recent theories have provided evidence regarding neurophysiological and autonomic responses post application of manipulative therapy [72-75]. Evidently, further research in this area is needed.

The protocol of therapeutic interventions may be another factor to consider in evaluating the results of the current study. Although there is no suggested regime on the number of repetitions that would benefit LBP patients, Mulligan [13] suggests the application of 3 sets of mobilisation each consisting of at least 4–6 repetitions; so this number of sets was adopted for this study. Furthermore, Kostantinou et al [11] found that most physiotherapists utilizing lumbar SNAGs mobilised two lumbar levels using two to three sets of 4–5 mobilisations in each lumbar level. Further repetitions may be performed depending on the severity, irritability and nature of the pathology and as participants were asymptomatic, 10 as opposed to 6 repetitions were adopted in this study since irritability was not an issue. Despite this increase in number of repetitions no significant change was observed and is therefore unlikely to be a contributory factor to the results.

The use of an asymptomatic population remains a potential limitation to this study. A sample of LBP patients may have been more appropriate in reflecting clinical practice. With symptomatic subjects, it is hypothesized that MWMs reduce pain and subsequently restore ROM through various inter-acting mechanisms including articular, peri-articular, neurophysiological, endocrine, and psychological effects [76,77]. Reduction in pain levels with MWMs could potentially decrease muscle spasm and thus facilitate movement thereby mitigating the chance of seeing ROM differences in asymptomatic subjects. A second limitation concerns the Zebris device used for measuring ROM. Although, this device is an efficient and easily applicable measuring tool, it has some stabilization problems as far as the two belts used to attach the device to the subjects' body are concerned. It is suggested that a fixation system similar to that used by Troke et al [78], where a plastic frame is stuck on subjects' skin, would be more appropriate for these measurements. Despite efforts to fix and standardise the position of the two belts (by securing them with tape and permanent ink markings), it is not unreasonable to assume that in the presence of an excellent fixation system, measurement errors (i.e. SEM and SDD values in Table 5) would be further minimised. Although the reliability study undertaken demonstrated very good within-day reliability however, this consideration cannot be underestimated. Another limitation involves the lack of standardisation of the manual force applied with the SNAG technique. The manual therapist applied a substantial amount of force to all subjects based on her clinical experience and judgment. However, as it happens with most studies involving manual therapy techniques, this force was not objectively measured or quantified. Although previous research has investigated mobilisation forces and the therapist's reliability [79], no study has ever explored the manual forces exerted during an MWM or the manual therapist's reliability of force application. Thus, the therapist's quantification and reliability of force applied with an MWM could be a point that merits further consideration in future studies.

In terms of clinical significance of the study, it is emphasized that ROM measurement may be a useful indicator of monitoring patient progress  [17-22,80,81] although it is argued that ROM solely has limited applicability whereas, more dynamic trunk motion indicators (such as velocity and acceleration) during exertion of movement or functional tasks have provided more sensitive information and can be used as a tool to track patient progress during treatments [18,82]. However, the need for further research involving symptomatic patient groups that takes into account functional outcome measures and neurophysiological changes (e.g. pain, sympathetic nervous system activity via e.g. skin conductance) is required to evaluate more fully the potential of MWMs in a clinical situation.

Regarding the reliability part of the study, the results demonstrated excellent intra- (ICCs = 0.89–0.90) and inter-day (ICCs = 0.82) reliability in lumbar measurements with the Zebris. Intra-day flexion measurements are comparable with previous reports, and in most cases our reliability is considerably higher with lower measurement errors [31,33,83-85]. Utilising the CA-6000 Spine Motion Analyzer, Shuit et al. [33] found ICC and SEM values of 0.95 and 3.7°, respectively in symptomatic subjects. Barret et al. [39] measured combined movements (including flexed positions) in LBP patients by utilizing Fastrack Polhemus system, and reported ICCs of 0.79–0.93. In a study comparing the CA-6000 Spine Motion Analyzer with the Fastrak Polhemus system, ICCs for flexion measurements were between 0.82–0.99 for both equipment [31]; however, SEM values were not reported. For inter-day measurements reliability for CA-6000 ICC was found 0.81 with SDD of 9.8% [78]. Although there are only a few published reports to compare with, our results are similar to Mannion et. al. [31], with an ICC over 0.80 and a SEM of 3.0° indicating that the Zebris can provide clinically relevant changes in ROM measurements following a therapeutic technique. Although discrepancies between published reports are small, the lack of reporting SEM and SDD values in most studies precludes comparisons, as reliability cannot be thoroughly evaluated without these reliability indices. Nevertheless, our results indicate good reliability and acceptable clinical applicability when measuring lumbar flexion with the Zebris system.

## Conclusion

In this prospective randomized double-blinded study, SNAG mobilizations applied at two lumbar levels in asymptomatic subjects did not demonstrate significant differences in the lumbar flexion ROM, when compared to a placebo group. Focus for further research should involve symptomatic subjects with reported (and/or measurable) restriction in lumbar ROM, and explore the global effect of MWMs in terms of biomechanics and pain. In terms of the reliability of the measuring equipment, although the present procedure yielded excellent intra- and inter-day reliability measurements, indicating good clinical applicability, improvement of the Zebris fixation system is recommended.

## Competing interests

The authors declare that they have no competing interests.

## Authors' contributions

MM carried out the ROM measurements in the interventional component of the study, was involved in the acquisition and interpretation of data, and wrote the first draft of the manuscript. EB served as project lead, was responsible for the conception and design, carried out the manual interventions, contributed in the analysis and interpretation of the interventional component and has been involved in drafting the manuscript. NS contributed substantially to the conception of the reliability component of the study, its data acquisition and analysis and was involved in drafting the manuscript. KP carried out the acquisition, analysis and interpretation of data in the reliability component of the study. JO helped in revision of the manuscript.

## Pre-publication history

The pre-publication history for this paper can be accessed here:


